# La lucha por la alimentación en medio de la inequidad social y la violencia: inseguridad alimentaria en el piedemonte amazónico en Caquetá

**DOI:** 10.7705/biomedica.7531

**Published:** 2025-09-22

**Authors:** Santiago Estrada, Michael Pasaje, Juan Pablo Botache, Fabián Méndez

**Affiliations:** 1 Grupo Epidemiología y Salud Poblacional, Universidad del Valle, Cali, Colombia Universidad del Valle Grupo Epidemiología y Salud Poblacional Universidad del Valle Cali Colombia

**Keywords:** abastecimiento de alimentos, población rural, conflictos armados, Food supply, rural population, armed conflicts

## Abstract

**Introducción.:**

El piedemonte amazónico es un territorio de contrastes entre la abundancia natural y las diversas problemáticas socioambientales. Las comunidades étnicas y campesinas -y, en particular, las mujeres- han sido las principales víctimas de estos conflictos, lo que ha exacerbado las brechas de desigualdad que le impiden a estas comunidades alcanzar niveles adecuados de bienestar, incluyendo la seguridad alimentaria.

**Objetivos.:**

Determinar la prevalencia de la inseguridad alimentaria e identificar los factores socioeconómicos asociados en dos municipios del suroeste del departamento del Caquetá.

**Materiales y métodos.:**

Se desarrolló un estudio transversal para estimar la prevalencia de la inseguridad alimentaria en Curillo y San José del Fragua, según la Escala Latinoamericana y Caribeña de Seguridad Alimentaria. Se aplicaron 189 encuestas en hogares de dichos municipios.

**Resultados.:**

Solo el 23,3 % de los hogares declararon tener seguridad alimentaria, mientras que el 30,2 % tuvo inseguridad alimentaria moderada y grave. Se encontraron asociaciones estadísticamente significativas entre la inseguridad alimentaria y la afiliación al régimen de salud, el nivel de escolaridad y el autorreconocimiento como víctima del conflicto armado.

**Conclusiones.:**

En estas poblaciones campesinas, la alta prevalencia de inseguridad alimentaria se encontró asociada a condiciones socioeconómicas de vulnerabilidad. Los municipios estudiados presentaban una prevalencia de inseguridad alimentaria muy superior al promedio nacional, lo que refleja la marginalidad y la desigualdad relacionadas con el conflicto armado, las limitaciones de acceso a los mercados y el desplazamiento de la vocación agrícola. Estos factores coinciden con el aumento sostenido de la inseguridad alimentaria en el país, incluso después de la firma del Acuerdo de Paz.

## Abstract (en)

**Introduction.:**

The Amazon foothills are a territory of contrasts between natural abundance and various socio-environmental problems. Ethnic and peasant communities -and particularly women- have been the primary victims of these conflicts, which have exacerbated inequality gaps that prevent these communities from achieving adequate levels of well-being, including food security.

**Objectives.:**

To determine the prevalence of food insecurity and identify the socioeconomic factors associated with its occurrence in two municipalities of the Andean-Amazon piedmont: San José del Fragua y Curillo.

**Materials and methods.:**

We conducted a cross-sectional study to estimate the prevalence of food insecurity in communities of two municipalities in the southwest of Caquetá, using the *Escala Latinoamericana y Caribeña de Seguridad Alimentaria.* One hundred eighty-nine surveys were applied to households in the municipalities of Curillo and San José del Fragua.

**Results.:**

Only 23.3% of the households declared food security, while 30.2% experienced moderate and severe food insecurity. Statistically significant associations were found between food insecurity and affiliation to the healthcare system, level of education, and self-recognition as a victim of the armed conflict.

**Conclusions.:**

In these peasant populations, the high prevalence of food insecurity was associated with vulnerable socioeconomic conditions. The studied municipalities showed prevalence rates higher than the national average, reflecting marginalization and inequality related to the armed conflict, limited access to markets, and displacement of the agricultural vocation. These factors align with the country's sustained increase in food insecurity, even after the signing of the Peace Agreement.

La alimentación ha desempeñado un papel vital en el desarrollo de las sociedades a lo largo de la historia y fue reconocida como un derecho fundamental en la Declaración Universal de los Derechos Humanos de 1948 ^1^. A pesar de ello, el hambre persiste como un problema grave que cada vez afecta a un mayor número de personas. En consecuencia, la erradicación del hambre se ha incluido entre los Objetivos de Desarrollo Sostenible ^2^. En la década de 1970, surgió el concepto de *seguridad alimentaria* como respuesta a la necesidad de evaluar la situación. La Cumbre Mundial sobre la Alimentación ^3^, la definió como el acceso físico, social y económico a alimentos suficientes, inocuos y nutritivos que satisfagan las necesidades energéticas diarias y las preferencias alimentarias para llevar una vida activa y sana. Esta definición fue confirmada en la Cumbre Mundial sobre la Alimentación del 2009, en la que, por primera vez, se hizo referencia a los cuatro pilares fundamentales de la seguridad alimentaria: disponibilidad, accesibilidad, estabilidad y utilización biológica de los alimentos. Asimismo, en la declaración final de dicha cumbre se enfatizó la dimensión nutricional como un componente esencial para garantizar la seguridad alimentaria en su totalidad ^4^.

Según el informe de la FAO, *El estado de la seguridad alimentaria y la nutrición en el mundo, 2023*^5^, el número de personas con inseguridad alimentaria aguda ha aumentado en 37 millones entre el 2021 y el 2022. Este fenómeno se ha exacerbado por el incremento de la inflación a nivel global, lo que ha generado un alza en los precios de los productos agrícolas, en particular de los cereales, y de las exportaciones. Asimismo, factores como los conflictos internacionales y las condiciones climáticas extremas -cada vez más frecuentes- ^6^ han contribuido significativamente a agravar la crisis alimentaria. A estos elementos se suman los efectos económicos y sociales adversos derivados de la pandemia de COVID-19 en todo el mundo ^7^^,^^8^, incluida Latinoamérica y el Caribe, donde el problema se ha agudizado por las catástrofes naturales y las deficiencias fiscales en la gestión de la crisis ^4^^-^^8^.

La publicación *Puntos calientes del hambre* identificó factores clave que aumentan la vulnerabilidad alimentaria, como la violencia organizada, los conflictos armados, los peligros naturales, las enfermedades zoonóticas, la economía y las limitaciones del acceso humanitario ^9^. Particularmente, los conflictos armados, además de causar tragedias humanitarias, están directamente relacionados con la inseguridad alimentaria y el hambre ^10^. Se ha señalado que más de la mitad de las personas subalimentadas y casi el 80 % de los niños con retraso del crecimiento viven en los países con algún tipo de conflicto, violencia o fragilidad ^11^. Estos conflictos ocurren principalmente en las áreas rurales y afectan directamente la actividad agrícola al perturbar los sistemas alimentarios, reducir las poblaciones rurales, destruir infraestructuras, debilitar la resiliencia y aumentar la vulnerabilidad alimentaria ^12^. Esto interrumpe el acceso a los mercados y aumenta los precios de los alimentos, lo que repercute negativamente en el desarrollo individual y colectivo; también, afecta la producción de bienes y servicios, lo que causa daños sociales importantes a largo plazo en estas regiones. Por ejemplo, la malnutrición infantil puede provocar discapacidades físicas y cognitivas de por vida ^10^.

Colombia tiene un potencial importante para la producción de alimentos debido a su favorable ubicación geográfica y a sus tierras productivas ^13^. Sin embargo, el conflicto armado -en el que participan diversos grupos ilegales- y la sistemática exclusión política, social y económica de las comunidades rurales del país, afectan el uso y la tenencia de la tierra, lo que dificulta la producción y el acceso a los alimentos ^14^. Este problema es particularmente grave en las poblaciones vulnerables, como las víctimas del conflicto armado, los hogares encabezados por mujeres y aquellos en los que el jefe del hogar tiene un bajo nivel educativo, los indígenas y los afrodescendientes, quienes tienen menos acceso a los alimentos ^15^^,^^16^.

De acuerdo con la Encuesta Nacional de la Situación Nutricional - ENSIN ^16^, el 54,2 % de los colombianos experimenta inseguridad alimentaria; las zonas rurales son las más afectadas, especialmente aquellas más apartadas. En estas regiones, la prevalencia de inseguridad alimentaria es casi 10 puntos porcentuales más alta (64,1%) que el promedio nacional ^17^. Además, los niveles de obesidad en la mayoría de los departamentos del sur del país - donde predominan las poblaciones altamente rurales y dispersas- superan el promedio nacional (18,7 %). Los datos anteriores reflejan la vulnerabilidad de la población rural colombiana a la inseguridad alimentaria y sus consecuencias, incluyendo la doble carga de desnutrición y obesidad ^18^.

Históricamente, las zonas rurales han sido impactadas por las principales problemáticas del país, relacionadas con la inequidad social asociada a la pobreza, el abandono estatal, la falta de acceso a los servicios públicos, la explotación de la tierra para grandes proyectos agroindustriales y mineros, el conflicto armado y los grupos ilegales que ejercen el control territorial. Además, en estas zonas se concentra la mayor parte de las comunidades campesinas y pueblos étnicos del país ^17^^,^^18^. Según la ENSIN ^16^, el 55,5 % de los habitantes del departamento del Caquetá experimentó algún grado de inseguridad alimentaria, 24,5 % de ellos, de moderada a grave. El más reciente resumen ejecutivo del Programa Mundial de Alimentos de la ONU (PMA) para Colombia ^15^ reportó que la inseguridad alimentaria moderada o grave afecta entre el 30,1 y el 40 % de los hogares del departamento.

El departamento de Caquetá, ubicado en la Amazonía colombiana y adyacente a la cordillera oriental, representa el 7,8 % del territorio nacional y el 22 % de la región amazónica del país. Su historia ha estado marcada por la economía extractivista, la violencia y los procesos de colonización dirigida, así como por la consolidación de la economía regional de la coca ^18^. A pesar del potencial agrícola de la región, el uso del suelo ha sido limitado por factores como el conflicto armado, la expansión ganadera, la minería, los cultivos ilícitos y la deforestación ^19^, lo que, sumado a la baja competitividad y a la falta de presencia institucional ^20^, ha generado desigualdad y migración de los campesinos hacia las zonas urbanas ^19^^-^^25^.

El asentamiento de los grupos armados ilegales en el Caquetá ha tenido una carga política e histórica importante para el conflicto social en este territorio porque ha perpetuado la violencia. Durante la década de 1990 y principios de la del 2000, la consolidación de grupos armados insurgentes -como las FARC-EP, el EPL y el M-19- y paramilitares, causó una crisis humanitaria grave por desplazamiento forzado en la región ^23^^,^^24^. El conflicto armado ha transformado el uso del territorio con la intensificación de la violencia contra los ecosistemas y los campesinos ^21^^,^^22^. El desplazamiento forzado y el despojo han contribuido a la ruptura del tejido social y han creado brechas entre la población y el estado. Esto ha reforzado la concentración de la riqueza y profundizado la desigualdad en las zonas rurales ^27^^-^^27^, al tiempo que ha provocado la fragmentación de las organizaciones campesinas ^22^^-^^28^.

Los municipios de Curillo y San José del Fragua, ubicados en el suroccidente del departamento de Caquetá, han experimentado una trayectoria marcada por la violencia y las fronteras extractivas vinculadas a las economías de enclave ^29^. Curillo, fundado en 1985, fue uno de los últimos municipios en consolidarse dentro del contexto de las bonanzas económicas como un puerto estratégico para las actividades extractivas de la Amazonía ^19^. Actualmente, el 22,6 % de su población tiene necesidades básicas insatisfechas, la mayoría en la zona rural. El índice de pobreza multidimensional del municipio es del 55,4 %: 50,2 % en la cabecera y 68,0 % en los centros poblados y las áreas rurales dispersas ^30^.

Por su parte, San José del Fragua se encuentra en la zona limítrofe con los departamentos del Cauca y Putumayo y ha estado históricamente vinculado a economías ilícitas y a crisis profundas derivadas del conflicto armado. Durante la época del paramilitarismo, esta región fue gravemente afectada, lo que generó desplazamientos masivos y alteraciones en la estructura social y económica del territorio ^29^.

Ambos municipios presentan altos índices de necesidades básicas insatisfechas y una concentración significativa de su población en zonas rurales, especialmente en las zonas de poblamiento disperso. Además, constituyen una de las principales áreas de transición del piedemonte amazónico ^31^, lo que les confiere una relevancia particular en términos ecológicos, sociales y económicos.

Desde la implementación del Acuerdo de Paz en Colombia, en el 2016, el departamento de Caquetá, específicamente los municipios de San José del Fragua y Curillo, han sido identificados como regiones prioritarias para dicha implementación y por ello tienen Programas de Desarrollo con Enfoque Territorial (PDET). Estos programas plantean una planeación y gestión a diez años, ya que estos territorios se encuentran entre los más afectados por el conflicto armado, presentan mayores índices de pobreza, presencia de economías ilícitas y debilidad institucional ^32^.

El objetivo de esta investigación fue determinar la prevalencia de la inseguridad alimentaria en San José del Fragua y Curillo, dos municipios rurales del suroccidente del departamento de Caquetá ubicados en el piedemonte amazónico, e identificar los factores socioeconómicos asociados a este fenómeno que afecta la salud y el bienestar de las comunidades.

## Materiales y métodos

## Tipo de estudio

Se realizó un estudio transversal para estimar la prevalencia de inseguridad alimentaria y explorar su asociación con algunas características demográficas y sociales de las familias de algunas comunidades de dos municipios del suroeste del Caquetá: San José del Fragua y Curillo.

La inseguridad alimentaria es un problema multidimensional que no puede ser medido directamente por una sola variable ^33^ y, por esta razón, se han desarrollado diversos instrumentos, como la Escala Latinoamericana y Caribeña de Seguridad Alimentaria (ELCSA). Este instrumento se basa en las experiencias de los hogares y se ha aplicado con éxito en Colombia, México y Brasil. La medición de la seguridad alimentaria ha sido ampliamente adoptada ^25^^,^^26^^,^^32^^,^^34^ y utilizada como herramienta para la formulación de políticas públicas ^35^.

**Figura 1:**
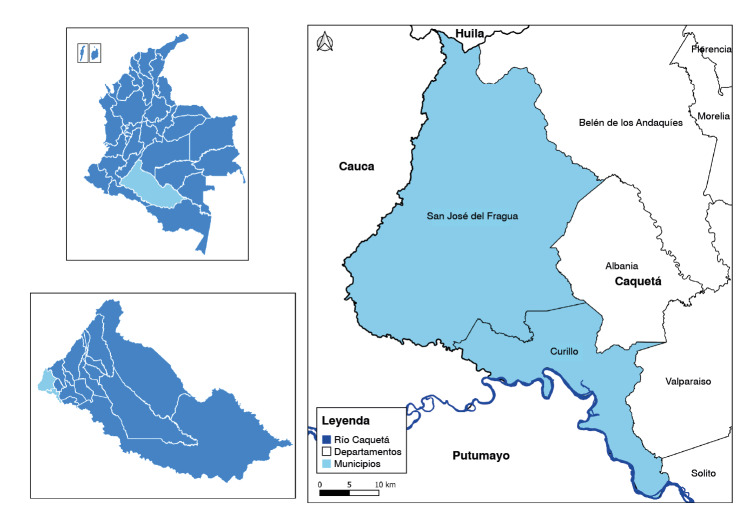
Área de estudio: Curillo y San José del Fragua, municipios del piedemonte amazónico en el departamento del Caquetá, Colombia

Mapa elaborado por los autores a partir de la infraestructura geoespacial del Instituto Geográfico Agustín Codazzi.

## Área y población

El estudio se llevó a cabo en los municipios rurales de San José del Fragua y Curillo, localizados en el piedemonte amazónico, al suroccidente del departamento de Caquetá (figura 1). La población de la región es predominantemente mestiza, con presencia de comunidades afrodescendientes e indígenas.

Estos municipios se caracterizan por una diversidad de paisajes fisiográficos debido a su ubicación en el piedemonte andino-amazónico, que van desde las montañas estructurales erosionadas que forman la vertiente de la cordillera oriental, hasta el piedemonte aluvial y el paisaje de lomerío amazónico ^36^, con alturas que oscilan entre los 200 y los 1.000 msnm. Sus suelos son fértiles y aptos para la agricultura y la ganadería, las principales actividades económicas de la región.

Los municipios poseen una gran riqueza de recursos naturales y culturales, pero en su desarrollo y conservación se enfrentan a retos importantes, como la deforestación, la minería ilegal, los cambios agresivos en la cobertura del suelo, el aumento de la "praderización" (conversión de áreas naturales a pastizales) y el conflicto armado.

### Muestra

El tamaño muestral se determinó para estimar la prevalencia de inseguridad alimentaria por hogar en el área de estudio. Se consideró una prevalencia esperada del 40 al 55 % con un margen de precisión del 10 %. Con base en estos parámetros, el tamaño calculado fue de 92 a 96 hogares.

Como se planeó estratificar las estimaciones por cada uno de los dos municipios, el total del tamaño de muestra se estableció entre 184 y 192 hogares. Además, debido a la situación de conflicto armado latente en el territorio, la selección de los hogares se hizo tratando de abarcar la mayor extensión posible del área de estudio, pero asegurando la movilidad y la seguridad del equipo encuestador.

### Procedimientos

Este estudio se desarrolló en el marco de una encuesta poblacional más grande que incluyó, además de las preguntas sobre seguridad alimentaria de la ELCSA, información de las familias sobre características sociodemográficas (por ejemplo, tamaño de la familia, edad, ocupación, nivel educativo, autorreconocimiento étnico, jefatura del hogar, régimen de salud de la jefatura del hogar), acceso a servicios de salud y agua potable, condiciones de saneamiento e higiene, y una sección específica que profundizó sobre aspectos de soberanía alimentaria.

Para determinar el nivel de seguridad alimentaria y los diferentes niveles de inseguridad se utilizó la ELCSA, compuesta por 15 preguntas relacionadas con la percepción de las personas sobre el acceso a los alimentos. Las primeras ocho van dirigidas a los adultos en los hogares y las 7 restantes, a los menores de 18 años ^37^. Además, dado el contexto campesino y de conflicto armado de la región, para el análisis se incluyeron las variables de acceso a tierras para uso agropecuario, extensión de la tierra y autorreconocimiento como víctima del conflicto armado.

La encuesta se ajustó mediante una prueba piloto para asegurarnos de que tuviera un lenguaje claro para la población objetivo. Los encuestadores fueron profesionales del equipo investigador entrenados en la aplicación del instrumento. El control de calidad se hizo durante el trabajo de campo. Se utilizó una versión digitalizada del cuestionario para ingresar los datos en línea y disminuir errores en el procesamiento de la información.

Esta investigación se ajustó a los principios de ética vigentes en Colombia y recibió aval del Comité de Ética de Investigación en Salud de la Universidad del Valle (Aval No. 035-022). Todos los participantes leyeron y firmaron un consentimiento informado, en el cual se explicó la finalidad de la encuesta, la voluntad de la participación, el anonimato y la confidencialidad de la información recolectada.

### Análisis estadístico

El procesamiento y análisis de los datos se ejecutó en el programa estadístico Stata™, versión 17.0. Se estimó la prevalencia de seguridad alimentaria y de los distintos niveles de inseguridad alimentaria (leve, moderada y grave), según la ELCSA, considerando además la presencia o ausencia de menores de edad en el hogar.

Luego del análisis exploratorio y descriptivo, se aplicó un análisis univariado para determinar la asociación entre la presencia de seguridad o inseguridad alimentaria y cada uno de los siguientes factores de exposición de interés: tamaño del hogar, autorreconocimiento étnico, jefatura del hogar, escolaridad, afiliación a servicios de salud, forma de adquisición de alimentos, acceso y tenencia de la tierra y autorreconocimiento como víctima del conflicto.

Finalmente, se construyeron modelos de regresión múltiple para estimar razones de prevalencia ajustadas mediante la regresión de Poisson con varianzas robustas (sic) *(robust).* Para estos análisis se realizó una categorización de los resultados de la ELCSA en las siguientes dicotomías:

1. seguridad alimentaria versus inseguridad alimentaria;
2. seguridad alimentaria e inseguridad alimentaria leve versus inseguridad alimentaria moderada y grave, y
3. seguridad alimentaria e inseguridad alimentaria leve y moderada versus inseguridad alimentaria severa.

Lo anterior, se realizó con el propósito de explorar los factores asociados a las diferentes categorías de intensidad de inseguridad alimentaria, que van desde cualquier nivel hasta únicamente la categoría grave. Aunque los modelos se interpretaron dando mayor certidumbre a las razones de prevalencia con un nivel de significancia estadística del 95 % o mayor, se resaltan también aquellos hallazgos con valores de p menores de 0,10 o 0,20, considerando el tamaño de muestra relativamente pequeño de este estudio.

## Resultado

Entre mayo y junio del 2023, se aplicaron 189 encuestas: 101 en Curillo y 88 en San José del Fragua. Las características de la población encuestada se describen en el cuadro 1.

En esta región de la Amazonía colombiana, cerca del 80 % de la población se autorreconoción como mestiza o blanca, el porcentaje restante se identificó como indígena o afrodescendiente; este último grupo representado mayoritariamente por una comunidad migrante del Pacífico asentada en el municipio de Curillo. Las características sociodemográficas de las familias indicaron condiciones de vulnerabilidad por los bajos niveles educativos, la ocupación en trabajo campesino e informal y la afiliación predominante al régimen subsidiado de salud. Aunque el 98 % de los hogares se identificó como campesino, la mitad de ellos poseía 10 hectáreas o menos y el 81 % no tenía títulos de propiedad.

**Cuadro 1:** Características de la población encuestada en Curillo y San José del Fragua, Caquetá, Colombia, 2023

Municipio		Curillo (n = 101)	San José del Fragua (n = 88)	Total (n = 189)
Promedio de personas por hogar (RIQ)		4	3	3
Número de personas por hogar (%)				
	1-2	20,8	33,0	26,5
	3-4	46,5	50,0	48,1
	> 4	32,7	17,0	25,4
Autorreconocimiento étnico				
	Afrodescendiente	16,8	1,1	9,5
	Indígena	8,9	14,8	11,6
	Mestizo/blanco	74,3	84,1	78,8
Jefatura del hogar				
	Femenina	15,8	31,8	23,3
	Compartida	31,7	25,0	28,6
Ocupación actual, población < 18 años				
	Estudiante	73,5	69,7	72,1
	Trabajo campesino	4,5	6,7	5,3
	Ama/o de casa	0,6	4,5	2,0
	Trabajo informal	0,6	2,2	1,2
Ocupación actual, hombres ≥ 18 años				
	Estudiante	4,5	1,1	3,0
	Ama/o de casa	1,8	1,1	1,5
	Trabajo campesino	89,9	78,0	84,5
	Trabajo comunitario*	7,3	5,5	6,5
	Trabajo formal	0,9	9,9	5,0
	Trabajo informal	12,8	14,3	13,5
	Cesante/buscando empleo	1,8	2,2	2,0
Ocupación actual, mujeres ≥ 18 años				
	Estudiante	8,6	2,2	5,6
	Ama/o de casa	79,0	67,7	73,7
	Trabajo campesino	31,4	25,8	28,8
	Trabajo comunitario*	4,8	3,2	4,0
	Trabajo formal	4,8	6,5	5,6
	Trabajo informal	3,8	17,2	10,1
	Cesante/buscando empleo	2,9	7,5	5,1
Nivel de escolaridad jefatura masculina				
	Ninguno	5,7	2,6	4,4
	Primaria	64,2	68,4	65,9
	Secundaria	26,4	26,3	26,4
	Técnico/tecnológico	1,9	0,0	1,1
	Profesional	1,9	2,6	2,2
Nivel de escolaridad jefatura femenina				
	Ninguno	0,0	3,6	2,3
	Primaria	43,8	46,4	45,5
	Secundaria	56,3	39,3	45,5
	Técnico/tecnológico	0,0	7,1	4,5
	Profesional	0,0	3,6	2,3
Régimen de salud jefatura masculina				
	Contributivo	1,9	0,0	1,1
	Subsidiado	92,5	97,4	94,5
	Ninguno	5,7	2,6	4,4
	No sabe/no responde	0,0	0,0	0,0
Régimen de salud jefatura femenina				
	Contributivo	0,0	7,1	4,5
	Subsidiado	100,0	85,7	90,9
	Ninguno	0,0	0,0	0,0
Distancia promedio al principal centro poblado municipal		18,6 km	26,7 km	22,6 km
Personas con acceso a tierras para uso agropecuario		83,2	60,2	72,5
Extensión de la tierra (ha)		0-5: 26,2	0-5: 49,1	0-5: 35,0
		5-10: 14,3	5-10: 13,2	5-10: 13,9
		10-20: 15,5	10-20: 5,7	10-20: 11,7
		20-50: 21,4	20-50: 22,6	20-50: 21,9
		50-100: 16,7	50-100: 7,5	50-100: 13,1
		100-200: 3,6	100-200: 1,9	100-200: 2,9
		> 200: 2,4	> 200: 0,0	> 200: 1,5
Autorreconocimiento como víctima del conflicto armado		81,2	77,3	79,4
Seguridad alimentaria				
	Seguridad	21,8	25,0	23,3
	Inseguridad leve	52,5	39,8	46,6
	Inseguridad moderada	19,8	26,1	22,8
	Inseguridad grave	5,9	9,1	7,4

* La categoría de trabajo comunitario se refiere al trabajo no remunerado pero que contribuye a disminuir los gastos económicos del hogar, como la tarifa moderadora de agua para el mantenimiento de acueductos comunitarios, etc.

**Figura 2:**
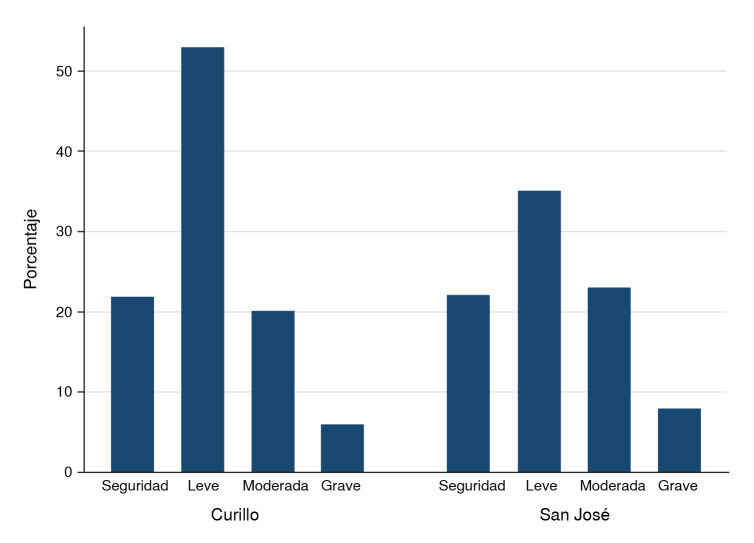
Prevalencia de seguridad e inseguridad alimentaria en los municipios de Curillo y San José del Fragua, Caquetá, Colombia

La figura 2 muestra la prevalencia de seguridad alimentaria y de los niveles leve, moderado y grave de inseguridad alimentaria. Solo el 23,3 % de los hogares declaró tener seguridad alimentaria, mientras que el 30,2 % manifestó niveles moderado y grave de inseguridad alimentaria, con una prevalencia estimada más alta en San José del Fragua (35,2 %) que en Curillo (25,7 %).

El cuadro 2 presenta los resultados de los modelos de regresión, las estimaciones de las razones de prevalencia de los análisis univariados y los resultados ajustados de la regresión múltiple. Los hallazgos estadísticamente significativos (p < 0,05) se relacionan con el tipo de afiliación al régimen de salud, el nivel de escolaridad y el autorreconocimiento como víctima del conflicto armado.

**Cuadro 2:** Factores asociados a la inseguridad alimentaria en San José del Fragua y Curillo en Caquetá, Colombia, 2023

		Modelo 1		Modelo 2		Modelo 3	
		Seguridad versus inseguridad alimentaria Razón de prevalencia (IC_95 %_)		Seguridad e inseguridad alimentaria leve versus inseguridad alimentaria moderada y grave Razón de prevalencia (IC_95 %_)		Seguridad e inseguridad alimentaria leve y moderada versus inseguridad alimentaria grave Razón de prevalencia (IC_95 %_)	
		Univariado	Regresión múltiple	Univariado	Regresión múltiple	Univariado	Regresión múltiple
Tamaño del hogar (número de personas)							
	1-2	1	1	1	1	1	1
	3-4	1,08 (0,87-1,34)	1,08 (0,87-1,35)	1,04 (0,58-1,88)	0,97 (0,54-1,76)	0,70 (0,23-2,09)	0,61 (0,20-1,84)
	> 5	1,17 (0,93-1,46)^φ^	1,18 (0,94-1,49)^φ^	1,36 (0,73-2,51)	1,17 (0,62-2,20)	0,38 (0,08-1,88)	0,33 (0,07-1,56)^φ^
Etnia							
	Mestizo	1	1	1	1	1	1
	Indígena/Afro	0,93 (0,76-1,15)	0,92 (0,75-1,12)	1,20 (0,74-1,97)	1,18 (0,72-1,96)	2,06 (0,73-5,81)^φ^	2,17 (0,81-5,80)^φ^
Jefatura del hogar:							
	Masculina o femenina	1	1	1	1	1	1
	Compartida	0,95 (0,80-1,14)	0,97 (0,80-1,17)	0,89 (0,54-1,47)	1,04 (0,63-1,71)	0,42 (0,10-1,81)	0,52 (0,10-2,60)
Régimen de salud							
	Subsidiado	1	1	1	1	1	1
	Contributivo	0,67 (0,42-1,06)^Ƴ^	0,69 (0,43-1,08)^φ^	0,18 (0,03-1,23)^Ƴ^	0,17 (0,02-1,17)^Ƴ^	0,03 (0,02-0,06)*	0,02 (0,00-0,06)*
Escolaridad del jefe de hogar							
	Ninguna	1	1	1	1	1	1
	Primaria	0,78 (0,70-0,87)*	0,80 (0,69-0,92)*	1,21 (0,44-3,32)	1,21 (0,43-3,44)	0,91 (0,12-6,63)	0,80 (0,09-6,97)
	Secundaria o superior	0,72 (0,62-0,82)*	0,75 (0,64-0,87)*	0,10 (0,35-2,79)	1,13 (0,41-3,39)	0,68 (0,09-5,32)	0,72 (0,79-6,53)
Acceso a tierras							
	No	1	1	1	1	1	1
	Sí	0,96 (0,81-1,14)	0,94 (0,79-1,11)	0,97 (0,60-1,58)	0,76 (0,46-1,24)	0,95 (0,31-2,90)	0,73 (0,26-2,04)
Víctima del conflicto armado							
	No	1	1	1	1	1	1
	Sí	1,19 (0,94-1,51)^φ^	1,16 (0,92-1,48)	2,70 (1,16-6,32)*	2,59 (1,14-5,85)*	3,37 (0,45-25,19)	3,99 (0,00-1,26)^Ƴ^

* p < 0,05

Ƴ p < 0,10

φ p < 0,20

Específicamente, la población afiliada al régimen contributivo tuvo un menor riesgo de tener inseguridad alimentaria a diferencia de aquellos con inseguridad alimentaria severa vinculados principalmente al régimen subsidiado. En relación con la escolaridad, los hogares cuyo jefe tenía estudios primarios o secundarios mostraron una menor prevalencia de inseguridad alimentaria en todos los niveles (22 y 28 % menos, respectivamente). Sin embargo, la escolaridad no parece tener un efecto protector en los niveles moderado o grave de inseguridad alimentaria. La prevalencia de inseguridad alimentaria moderada y grave fue 2,6 veces más frecuente en las familias que se reconocían como víctimas del conflicto armado, incluso después de ajustar por las demás variables en el modelo de regresión múltiple.

Por otra parte, aunque no se encontró una asociación estadísticamente significativa entre la inseguridad alimentaria y el tamaño del hogar, sí se evidenció una tendencia según la cual los hogares más numerosos presentaron mayor riesgo de inseguridad alimentaria, aunque en la categoría leve. Por el contrario, los hogares con cinco o más personas presentaron una menor prevalencia de inseguridad alimentaria grave. Otro hallazgo sugiere que la población autorreconocida como indígena o afrodescendiente tuvo una mayor prevalencia de inseguridad alimentaria grave, con un valor ligeramente superior al doble en los análisis univariados y de regresión múltiple (12,50 vs. 6,08; p < 0,20).

Además, vale la pena analizar dos hallazgos, a pesar de su falta de significancia estadística. En primer lugar, los resultados sugieren que los hogares con jefatura compartida tienden a tener una menor prevalencia de inseguridad alimentaria grave en comparación con aquellos que tienen una sola jefatura (ya sea masculina o femenina). En segundo lugar, la ausencia de un efecto protector relacionado con el acceso a la tierra fue consistente en los tres modelos (razones de prevalencia cercanas a 1). Esto indica que los determinantes de la inseguridad alimentaria en la ruralidad necesitan analizarse en el contexto de unas condiciones sociales y de vida que van más allá del mero acceso a la tierra.

## Discusión

El análisis de la seguridad alimentaria en estas poblaciones campesinas, víctimas del conflicto armado en el piedemonte amazónico del Caquetá, describe los altos niveles de inseguridad alimentaria y los principales factores asociados. Se encontró que la alta prevalencia de inseguridad alimentaria -en todos los niveles- está asociada a condiciones socioeconómicas que generan vulnerabilidad. Los municipios estudiados, de carácter predominantemente rural, presentaron una prevalencia de inseguridad alimentaria del 77 %, en promedio 22 % por encima de los porcentajes nacional (54,2 %) y departamental (55,5 %) según la última ENSIN ^16^. Estas cifras pueden ser el resultado de la marginalidad y la desigualdad relacionadas con el conflicto armado, las dificultades de acceso a los mercados y el desplazamiento de la vocación agrícola ^38^^-^^40^. Estos factores coinciden con el aumento sostenido de la inseguridad alimentaria en el país, incluso después de la firma del Acuerdo de Paz ^41^.

Diversos análisis en áreas de fronteras extractivas, como Caquetá, han mostrado cómo los hechos de violencia y los procesos complejos de victimización tienen múltiples repercusiones en la salud ^42^. En particular, la concurrencia de condiciones de empobrecimiento (violencia estructural), el trauma inducido por la guerra (violencia directa) y la exposición a contaminantes (violencia lenta) tienen como efecto amplificar la carga de la enfermedad entre poblaciones ya desfavorecidas.

Aunque se podría suponer que en las zonas rurales hay una mayor producción de alimentos para el autoconsumo, la inseguridad alimentaria puede presentarse cuando los recursos económicos son limitados. En poblaciones colombianas, se ha reportado que la principal estrategia de afrontamiento para mitigar la inseguridad alimentaria es consumir alimentos de menor calidad, más baratos, y pedir dinero prestado para comprar alimentos. Esto puede dar lugar a una dieta con una variedad limitada y porciones más pequeñas ^43^. En las zonas rurales, una estrategia documentada es la venta de activos como herramientas, animales y semillas que pudieran servir para las próximas cosechas o producciones ^43^.

La ausencia de un efecto protector del acceso a la tierra frente a la inseguridad alimentaria en estas poblaciones campesinas puede explicarse por el compromiso de los activos fundamentales necesarios para la producción de bienes y servicios futuros en las zonas rurales. Esto puede llevar a un ciclo de precarización de las zonas rurales, que inevitablemente resulta en inseguridad alimentaria. Los resultados de este trabajo apoyan estudios previos que han mostrado que la propiedad de la tierra por sí sola no garantiza la seguridad alimentaria en Colombia ^44^^,^^45^.

Algunos estudios han encontrado una mayor prevalencia de inseguridad alimentaria en familias con un mayor número de miembros ^39^^,^^40^^,^^44^. Lo anterior podría relacionarse con el hecho de que, en esas familias, las personas no hacen parte de la población económicamente activa y, por lo tanto, tienen mayor probabilidad de experimentar inseguridad alimentaria ^41^. Sin embargo, los datos de este estudio muestran una disminución de la inseguridad alimentaria moderada y grave en los hogares con más de cuatro miembros (razón de prevalencia = 0,33). Aunque la dependencia económica puede aumentar en las familias numerosas ^44^, el comportamiento observado en los hogares de la zona de estudio puede atribuirse a un aumento del número de miembros laboralmente activos, lo que tendría un efecto protector. Este efecto le permitiría a las familias afrontar la precariedad mediante diversas fuentes de recursos materiales e ingresos en un contexto rural de campesinos proletarizados por las actividades extractivas y la dependencia de economías ilícitas como la coca en la región.

Lo anterior se contextualiza en el surgimiento de la ruralidad en la Amazonía, resultado de una serie de bonanzas económicas que no lograron consolidar condiciones de vida estables en la región. Inicialmente, se destacó la explotación del caucho, seguida de la quina, las pieles, la madera, la coca y, paralelamente, la expansión de la ganadería. No obstante, la ausencia de políticas agrarias focalizadas en el territorio ha tenido un profundo impacto en las familias campesinas, en la dinámica de producción, el modo de vida y las dimensiones socioculturales de la ruralidad ^46^.

Los hallazgos del presente estudio indican una estrecha relación entre ser víctima del conflicto armado y la inseguridad alimentaria. Esta relación puede atribuirse a la trayectoria histórica del conflicto en este territorio, marcada por la presencia de la guerrilla, los grupos paramilitares y la ausencia del propio Estado, lo que ha impactado profundamente la estructura agraria y la economía local.

Según la Unidad de Restitución de Tierras ^29^, la población rural en Colombia ha experimentado por décadas altos índices de homicidios, desplazamientos, abandono y despojo. Los constantes ciclos de violencia han provocado que las comunidades se desplacen hacia áreas urbanas y abandonen sus territorios, lo que ha generado un fenómeno de acaparamiento de tierras ^28^. Además, el control territorial ejercido por los grupos armados al margen de la ley ha llevado al establecimiento de cultivos ilícitos como alternativa de subsistencia para las comunidades campesinas. Asimismo, se han establecido procesos de expansión minera, agroindustrial y de ganadería extensiva que han desplazado los cultivos tradicionales destinados al *pancoger* y a su comercialización. Esta situación ha transformado los modos de vida imponiendo al campesinado vinculación forzada a estas economías y remuneración precaria ^46^.

Una vez se establecen las prácticas de despojo en un territorio y comienzan las intervenciones estatales para combatir las economías ilícitas, los campesinos resultan aún más perjudicados. Por ejemplo, durante los procesos de erradicación forzada y fumigaciones de cultivos ilícitos, los campesinos se ven obligados a eliminar cualquier fuente de sustento que pueda favorecer la seguridad alimentaria en la región ^46^^,^^47^. Estos resultados coinciden con los hallazgos en otras partes del mundo afectadas por conflictos armados en zonas rurales, donde se reportan disrupciones en la producción agrícola y acceso limitado a los alimentos. Los sistemas alimentarios se ven alterados y destruidos, lo que reduce la resiliencia y aumenta la vulnerabilidad de las poblaciones ^12^^,^^48^.

El análisis del tipo de jefatura de hogar revela una relación entre género y alimentación. La jefatura del hogar compartida entre hombres y mujeres se asoció con menores niveles de inseguridad alimentaria, como se demostró en estudios previos que también aplicaron la ELCSA ^49^^,^^50^. Este comportamiento podría explicarse por un mayor número de personas que contribuyen al hogar, lo que impacta en la reducción de la inseguridad alimentaria y la brecha de género. Otras investigaciones indican que los hogares encabezados por mujeres experimentan una mayor prevalencia de inseguridad alimentaria debido a las desigualdades económicas, sociales, políticas y ambientales que persisten en los roles de género ^43^. Estas desigualdades restringen las oportunidades de subsistencia, educación y crecimiento de las mujeres, lo que se traduce en un acceso y control limitados a los recursos para satisfacer sus necesidades nutricionales ^43^^,^^51^. Sin embargo, es importante señalar que las mujeres cabeza de familia pueden tener mejores habilidades de gestión alimentaria y estrategias de afrontamiento más dinámicas en situaciones estresantes y adversas, a pesar de enfrentarse a las desigualdades mencionadas. En general, las mujeres cabeza de familia tienen menos probabilidades de experimentar inseguridad alimentaria grave en comparación con sus homólogos hombres ^41^^,^^43^^,^^49^.

En el desarrollo de este estudio, es importante considerar algunas limitaciones metodológicas y analíticas. Aunque la ELCSA es un instrumento validado y ampliamente utilizado, presenta restricciones para comprender mejor el contexto del acceso a los alimentos y determinar el nivel de seguridad alimentaria ^45^^,^^52^^,^^53^. Además, el presente estudio tiene limitaciones relacionadas con el tamaño de muestra y la ausencia de indicadores antropométricos que habrían podido fortalecer los hallazgos. En este análisis no se incluyó información sobre la calidad de la dieta en relación con los contextos culturales y socioeconómicos, enfoques complementarios que podrían ayudar a explorar la relación entre la alimentación y el género o la etnia ^54^^,^^55^.

Para el diseño e implementación de los planes de política alimentaria en territorios rurales con población campesina víctima del conflicto armado, resulta fundamental considerar los hallazgos de este estudio relacionados con los determinantes sociales de las inequidades en seguridad alimentaria. Esto se enmarca en lo establecido en el *Acuerdo general para la terminación del conflicto y la construcción de una paz estable y duradera,* donde se menciona que

"[...] las acciones que se diseñen y ejecuten tendrán en cuenta las necesidades, características y particularidades de los territorios y las comunidades rurales y contarán con una perspectiva de género y enfoque diferencial [...]." 56,57
